# Antioxidant Activity of Ethanolic *Litchi chinensis* Seed Extract in Oxidative Stress Model Mice and Identification of Blood-Entering Prototype Components

**DOI:** 10.3390/molecules31132233

**Published:** 2026-06-25

**Authors:** Li Zhang, Aicun Tang, Ziming Yang, Wei Li

**Affiliations:** 1 Engineering Research Center of Innovative Drug of Traditional Chinese and Zhuang Yao Medicine, Ministry of Education, Guangxi University of Chinese Medicine, Nanning 530200, China; zl@gxib.cn (L.Z.); tangaicun@126.com (A.T.); 2 Guangxi Key Laboratory of Plant Functional Phytochemicals and Sustainable Utilization, Guangxi Institute of Botany, Guangxi Zhuang Autonomous Region and Chinese Academy of Sciences, Guilin 541006, China; 3 Engineering Research Center of Medical Institution Preparations and Medicine-Food Homology Products, Guangxi Zhuang Autonomous Region, Guangxi University of Chinese Medicine, Nanning 530200, China

**Keywords:** Litchi seed, flavonoids, UHPLC-MS, blood-entering components, network pharmacology, molecular docking

## Abstract

*Litchi chinensis* seeds are rich in flavonoids and exhibit potent antioxidant activity. This study constructed a D-galactose-induced oxidative stress model in mice and applied ultra-high performance liquid chromatography–mass spectrometry (UHPLC-MS), network pharmacology, and molecular docking to clarify the antioxidant activity and material basis of ethanolic litchi seed extract. Litchi seed extract was orally given by gavage at 100 and 200 mg/kg in antioxidant tests, whereas a dosage of 500 mg/kg was adopted for the detection of absorbed constituents in plasma. The results showed that the total flavonoid content of litchi seed extract reached 68.37%. The extract could markedly reduce malondialdehyde (MDA) levels and elevate superoxide dismutase (SOD) activity in the serum, liver and kidney tissues of model mice, thereby mitigating oxidative damage. Thirteen prototype compounds absorbed into blood were characterized by UHPLC-MS. Most of these substances were flavonoids, with isorhamnetin, quercetin and naringenin as the major representatives. Core targets including *IGF1R*, *PIK3R1*, *EGFR*, *PIK3CA*, *ERBB2* and proto-oncogene tyrosine-protein kinase Src (*SRC*) were screened using network pharmacology, among which *SRC* was identified as the pivotal hub target. Molecular docking results revealed that isorhamnetin, quercetin, naringenin, and diosmetin were able to bind stably to the SRC protein. The present study demonstrated that litchi seed extract exhibits remarkable antioxidant activity, with isorhamnetin, quercetin, naringenin, and diosmetin as the main bioactive antioxidant components.

## 1. Introduction

Litchi seed is the dried ripe seed of *Litchi chinensis* Sonn. It is widely cultivated in Guangdong, Guangxi, Yunnan, Sichuan, and other regions in China. As a Traditional Chinese Medicine (TCM), it has been officially recorded in all editions of the *Pharmacopoeia of the People’s Republic of China*. In TCM theory, Litchi Semen is warm in nature, sweet and slightly bitter in flavor, and channels to the liver and kidney meridians. It exerts the effect of dispelling cold and relieving pain. Clinically, it is commonly used for the treatment of hernial pain and testicular swelling and pain, and has been widely employed in the prevention and treatment of relevant diseases in TCM clinical practice for thousands of years [1].

The litchi tree is indigenous to China, which ranks as the world’s largest producer, accounting for over 50% of global output [2]. During litchi processing, litchi seeds account for approximately 25% of fresh fruit weight and are mostly discarded as waste. Therefore, reducing litchi postharvest losses and improving the utilization efficiency of litchi seeds have become urgent problems to be solved for the sustainable development of the litchi industry.

Literature surveys have demonstrated that litchi seeds are abundant in diverse bioactive constituents [3]. Multiple phytochemicals have been isolated and identified from litchi seeds, including gallic acid, procyanidin B2, epicatechin, quercetin, epicatechin-3-gallate, coumaric acid, protocatechuic acid, diosmetin, narirutin, cinnamtannin B1, naringin, pinocembrin-7-neohesperidoside, litchitannin A1, and litchitannin A2. Among these compounds, flavonoids constitute the predominant components [3,4].

Flavonoids constitute an important class of oxygen-containing heterocyclic organic compounds with potent antioxidant properties [5]. Accumulating evidence has indicated that litchi seeds exert prominent antioxidant effects both in vitro and in vivo [6,7]. In vitro pharmacological assays have demonstrated that the *n*-butanol and ethanol fractions of litchi seeds possess strong 1,1-diphenyl-2-picrylhydrazyl (DPPH) radical-scavenging capacity [8,9]. Litchi seed fermentation broth can be developed as a cosmetic raw material, which exhibits excellent antioxidant and anti-aging effects on human skin with good biosafety [10]. A high-fat diet supplemented with litchi seed powder markedly alleviated obesity in zebrafish by ameliorating oxidative stress [11]. Oxidative stress is defined as a redox imbalance in vivo, characterized by the excessive production of reactive oxygen species (ROS) alongside inadequate antioxidant scavenging capacity [12,13]. Despite the documented antioxidant activity of certain monomeric compounds in litchi seeds, the complex interplay among its multiple constituents may lead to substantially different effects compared with individual compounds. Accordingly, the present study was designed to investigate the antioxidant activity of litchi seed extract and clarify the material basis underlying its antioxidant effects in mice with D-galactose-induced oxidative stress.

Guided by the serum pharmacochemistry theory of TCM, the actual bioactive components responsible for the therapeutic effects of litchi seed extract are usually inconsistent with its intrinsic constituents. Only by isolating and identifying the in vivo effective constituents that directly function in blood can we accurately elucidate the pharmacodynamic material basis underlying the antioxidant activity of litchi seed extract [14]. Notably, a small number of components may also exert their pharmacological effects by regulating gut microbiota. Mass spectrometry, sensitive and capable of multi-stage fragmentation, is critical for phytochemical identification [15]. Untargeted scanning and dynamic exclusion enhance the detection of trace components to clarify extract chemical profiles [16]. In this study, ultra-high performance liquid chromatography–mass spectrometry (UHPLC-MS) was used for the qualitative analysis of chemical constituents in litchi seeds. An oxidative stress model was established in C57 mice by intraperitoneal injection of D-galactose. After verifying successful model establishment, the model mice were treated with litchi seed extract. Subsequently, blood samples were analyzed by UHPLC-MS combined with component profiling of litchi seeds to identify prototype constituents absorbed into the systemic circulation. This study aims to provide reliable evidence for clarifying the pharmacodynamic material basis underlying the antioxidant effects of litchi seeds.

Network pharmacology matches the holistic traits of herbal extracts via multi-target analysis, yet its application suffers from rigid frameworks and homogeneous predictions. Quercetin, kaempferol, and β-sitosterol are repeatedly identified as core active compounds across herbal disease studies, which lack scientific rationality [17,18]. This bias originates from database-dependent component acquisition, as public datasets cannot reflect the actual phytochemical profiles of experimental samples. To address the above-mentioned problems, appropriate revisions were made to the application of network pharmacology in exploring the antioxidant material basis of litchi seeds. The identified blood-entering prototype components of litchi seed extract were taken as research subjects, and network pharmacology was used to screen the major effective ingredients with antioxidant activity.

## 2. Results

### 2.1. Total Flavonoid Content in Litchi Seed Extract

Table 1 demonstrates that the total flavonoid content of the litchi seed extract reached 68.37% of the total extract weight, which quantitatively confirms that flavonoids represent the most abundant chemical class and predominant constituents in the prepared extract. Previous studies have confirmed that flavonoids possess potent antioxidant activity [5], suggesting that the obtained litchi seed extract may exert potential antioxidant effects.

### 2.2. In Vivo Antioxidant Activity of Litchi Seed Extract

**Figure 1:**
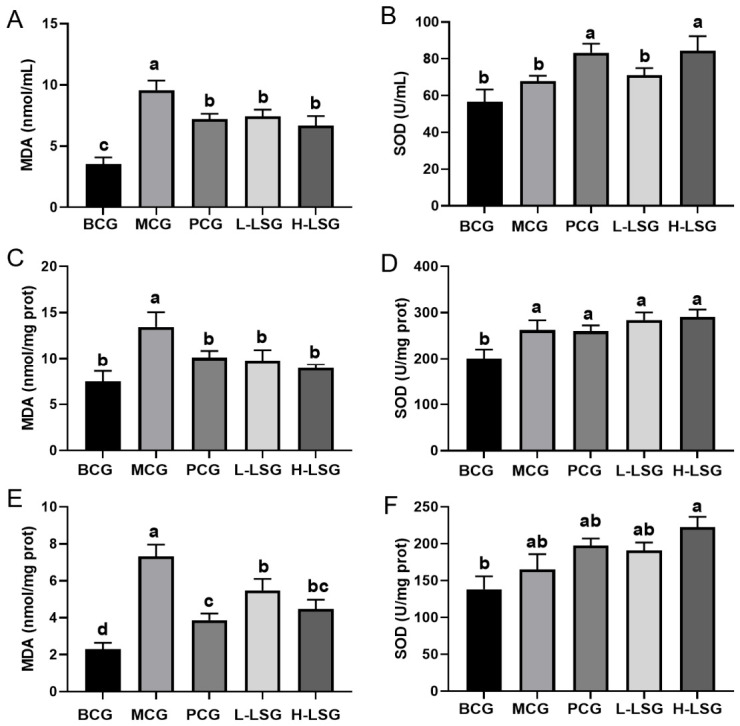
Effects of litchi seed extract on the systemic antioxidant capacity in mice. (**A**) Serum MDA content; (**B**) Serum SOD activity; (**C**) Liver MDA content; (**D**) Liver SOD activity; (**E**) Kidney MDA content; (**F**) Kidney SOD activity. Data are presented as mean ± standard deviation (*n* = 12 per group). Values marked with different lowercase letters (a, b, c) are significantly different at *p* < 0.05. Abbreviations: BCG, blank control group; MCG, model control group; PCG, positive control group; L-LSG, low-dose litchi seed extract group; H-LSG, high-dose litchi seed extract group; MDA, malondialdehyde; SOD, superoxide dismutase.

As shown in Figure 1A,C,E, compared with the blank control group (BCG), the malondialdehyde (MDA) levels in the serum, liver, and kidney of mice in the model control group (MCG) were significantly increased (*p* < 0.05). These results indicated that long-term 400 mg/kg D-galactose administration successfully induced oxidative stress in mice, which is consistent with previous findings [19]. After intervention with litchi seed extract, both the high- and low-dose (100 mg/kg and 200 mg/kg) showed markedly reduced MDA contents in serum, liver, and kidney relative to the MCG mice (*p* < 0.05). This suggests that litchi seed extract exerts a prominent protective effect against D-galactose-induced oxidative stress in mice. It is well documented that under oxidative stress, the body activates its antioxidant defense system by elevating the activities of endogenous antioxidant enzymes such as superoxide dismutase (SOD), thereby protecting cells from oxidative damage [13]. Consistent with this mechanism, our results revealed that SOD activities in the serum, liver, and kidney were higher in the MCG than in the BCG, with a significant difference observed in the liver (*p* < 0.05; Figure 1B,D,F). Moreover, intervention with litchi seed extract further increased SOD activity in mouse serum, liver, and kidney relative to the MCG. As shown in Figure 1, the positive drug vitamin E could reduce the MDA content and increase SOD activity in mice under oxidative stress. In kidney tissues, vitamin E exhibited a better effect on lowering MDA levels than the low-dose litchi seed extract (100 mg/kg). In blood samples, vitamin E was superior to the low-dose litchi seed extract in elevating SOD activity. In summary, mice can counteract D-galactose-induced oxidative damage by upregulating endogenous antioxidant enzyme activity. Furthermore, litchi seed extract effectively enhances antioxidant enzyme activities in vivo, thereby alleviating oxidative injury and exerting a protective effect on the organism.

### 2.3. Identification of Blood-Absorbed Prototype Constituents from Litchi Seed Extract

**Figure 2:**
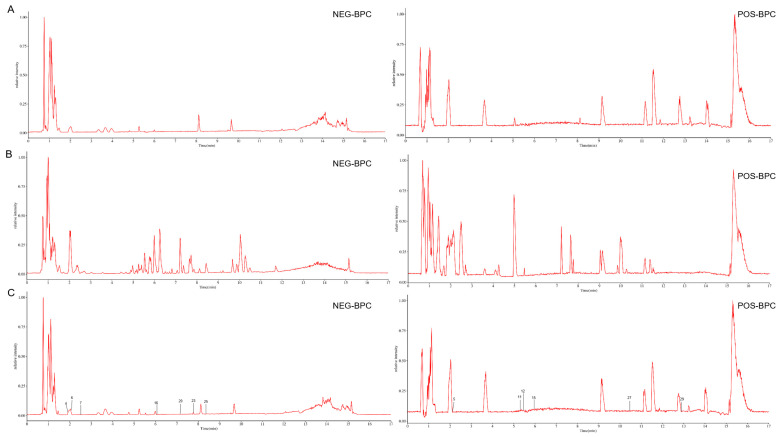
Total ion current (TIC) chromatograms of litchi seed extract. (**A**) Blank serum; (**B**) Litchi seed extract; (**C**) serum from mice treated with litchi seed extract. Abbreviations: NEG-BPC, positive mode base peak chromatogram; POS-BPC, negative mode base peak chromatogram.

**Figure 3:**
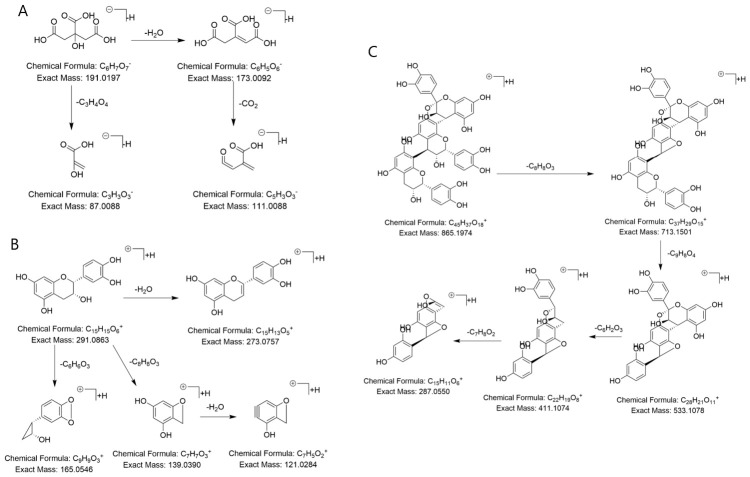
Proposed fragmentation pathways of representative compounds. (**A**) Fragmentation pathway of citric acid. (**B**) Fragmentation pathway of epicatechin. (**C**) Fragmentation pathway of cinnamtannin B1.

Distinct metabolic profiles were observed in serum samples from D-galactose-induced oxidative stress mice after litchi seed extract intervention, as characterized by UHPLC-MS analysis. By comparatively analyzing the chemical compositions of litchi seed extract and blank serum, prototype blood-derived components were successfully characterized, namely eight flavonoids, three organic acids, and two polyphenols. Detailed results are presented in Table 2 and Figure 2. The identification of blood-entering prototype components derived from litchi seed extract is exemplified by Compounds **5**, **7**, and **20**. Compound **5** possessed the molecular formula C_10_H_6_O_3_ with a retention time of 2.143 min. In negative ion mode, its quasi-molecular ion peak appeared at *m*/*z* 191.0197. The MS^2^ fragment ions were observed at *m*/*z* 173.0092 [M−H−H_2_O]^−^, *m*/*z* 111.0088 [M−H−H_2_O−CO_2_]^−^, and *m*/*z* 87.0088 [M−H−C_3_H_4_O_4_]^−^. Based on the fragmentation information, Compound **5** was tentatively identified as citric acid, a typical organic acid. Its proposed fragmentation pathway is presented in Figure 3A. Compound **7** had a molecular formula of C_15_H_14_O_6_ and a retention time of 2.463 min. In positive ion mode, the quasi-molecular ion peak was detected at *m*/*z* 291.0683. The corresponding MS^2^ fragment ions were *m*/*z* 273.0757 [M+H−H_2_O]^+^, *m*/*z* 165.0546 [M+H−C_6_H_6_O_3_]^+^, *m*/*z* 139.0390 [M+H−C_8_H_8_O_3_]^+^, and *m*/*z* 121.0284 [M+H−C_8_H_8_O_3_−H_2_O]^+^. Accordingly, Compound **7** was tentatively assigned as epicatechin, a flavonoid. Its fragmentation pathway is shown in Figure 3B. Compound **20** had a molecular formula of C_45_H_36_O_18_ and a retention time of 7.174 min. In positive ion mode, its quasi-molecular ion peak was observed at *m*/*z* 865.1794. The characteristic MS^2^ fragment ions appeared at *m*/*z* 713.1501 [M+H−C_8_H_8_O_3_]^+^, *m*/*z* 533.1078 [M+H−C_8_H_8_O_3_−C_9_H_8_O_4_]^+^, *m*/*z* 411.1074 [M+H−C_8_H_8_O_3_−C_9_H_8_O_4_−C_6_H_2_O_3_]^+^, and *m*/*z* 287.0550 [M+H−C_8_H_8_O_3_−C_9_H_8_O_4_−C_6_H_2_O_3_−C_7_H_8_O_2_]^+^. Accordingly, Compound **20** was tentatively identified as cinnamtannin B1, a polyphenol. The proposed fragmentation pathway is shown in Figure 3C.

### 2.4. Screening of Common Targets Between Blood-Entering Prototype Constituents of Litchi Seed Extract and Antioxidant Targets, and Construction of Component-Target Network

**Figure 4:**
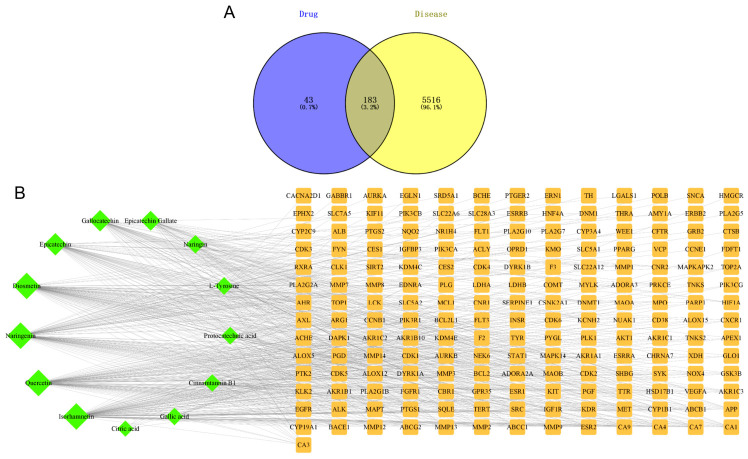
Screening of effective components associated with the antioxidant activity of litchi seed extract. (**A**) Venn diagram showing the intersection between targets of blood-entering prototype components of litchi seed extract and antioxidant-related targets; (**B**) network of blood-entering prototype components and their antioxidant targets.

All thirteen blood-entering prototype constituents of litchi seed extract were confirmed to have matched protein targets. A large pool of 5699 human genes closely linked to antioxidant biological processes was collected as background antioxidant-related targets. After matching the predicted targets of litchi seed-derived prototype components against this antioxidant gene set, 183 shared target genes were captured. These intersecting genes were presumed to be the key molecular targets mediating the antioxidant efficacy of litchi seed extract, and the matching relationship is intuitively displayed in the Venn diagram (Figure 4A).

Network visualization demonstrated comprehensive interactions among the 13 blood-absorbed prototype constituents from litchi seed extract and its 183 overlapping antioxidant targets (Figure 4B). In this network, node size represents the number of associated targets; a larger node indicates more interacting targets, suggesting that the corresponding compound possesses greater potential relevance to antioxidant regulation. Compounds with more interacting targets may play pivotal roles in mediating the antioxidant activity of litchi seed extract. Based on node size ranking, the top four core compounds were isorhamnetin, quercetin, naringenin, and diosmetin.

### 2.5. Construction of Protein–Protein Interaction (PPI) Network for Potential Antioxidant Targets of Litchi Seed Extract

**Figure 5:**
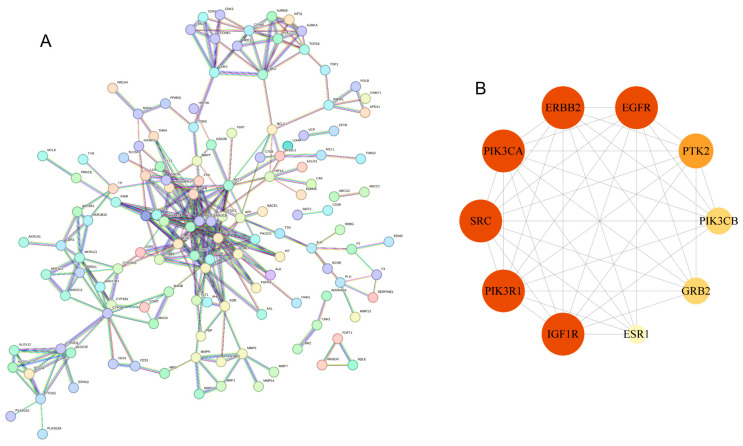
PPI network. (**A**) Network of intersection targets. (**B**) Network of core targets.

The 183 overlapping antioxidant targets of litchi seed extract formed an interconnected PPI system. The global PPI network contained 134 protein nodes connected by 310 interaction edges (Figure 5A). Further screening of hub proteins based on topological characteristics yielded a refined core target PPI network (Figure 5B). In this network, nodes with a deeper color represented proteins with more intensive interactions with other partner targets. Finally, six core antioxidant targets were identified: insulin-like growth factor 1 receptor (*IGF1R*), phosphoinositide-3-kinase regulatory subunit 1 (*PIK3R1*), proto-oncogene tyrosine-protein kinase Src (*SRC*), phosphoinositide-3-kinase catalytic subunit alpha (*PIK3CA*), erb-B2 receptor tyrosine kinase (*ERBB2*), and epidermal growth factor receptor (*EGFR*).

### 2.6. Molecular Docking Was Employed to Verify the Binding Interactions Between Active Ingredients and Core Targets

**Figure 6:**
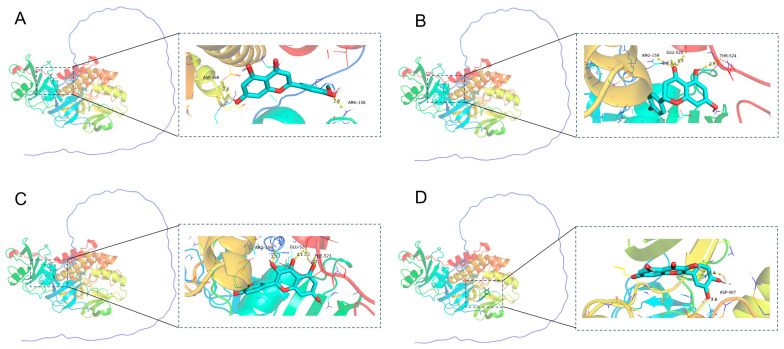
Molecular docking between SRC protein and compounds. (**A**) Diosmetin. (**B**) Naringenin. (**C**) Isorhamnetin. (**D**) Quercetin.

The six core antioxidant targets screened in Section 2.5 were *IGF1R*, *PIK3R1*, *SRC*, *PIK3CA*, *ERBB2*, and *EGFR*. Among these targets, *SRC* exhibits the closest association with antioxidant activity [20]. Therefore, molecular docking was performed between the SRC protein and the top four antioxidant-active compounds screened from litchi seed extract in Section 2.4, namely isorhamnetin, quercetin, naringenin, and diosmetin. This analysis aimed to verify the reliability of the screened active components and core targets. The docking results showed that the binding energies of all four candidate compounds with the SRC protein were lower than −7.0 kcal/mol, indicating that each component could spontaneously and stably bind to the active pocket of the SRC protein and exert potential targeted regulatory effects. The detailed binding characteristics of each compound to the SRC protein are presented in Table 3 and Figure 6. Diosmetin showed a docking binding energy of −7.3 kcal/mol. It formed stable polar interactions with the ASP-368 and ARG-158 residues in the active region of the SRC protein and anchored its molecular conformation via hydrogen bonding. Naringenin also had a binding energy of −7.3 kcal/mol and mainly interacted with ARG-159, GLU-520, and THR-524. The involvement of multiple polar residues effectively improved the binding stability of the ligand–protein complex. Isorhamnetin exhibited a binding energy of −8.2 kcal/mol and established multiple interactions with ARG-159, GLU-520, and PHE-523. Among these, hydrophobic interaction with PHE-523 further enhanced binding affinity. Quercetin had the lowest binding energy of −8.8 kcal/mol and displayed the strongest binding affinity among the four compounds. It formed strong polar interactions with the key functional residue ASP-407, enabling it to directly occupy the critical binding site of the SRC protein. Comprehensive evaluation of binding energy, key amino acid interaction sites, and spatial matching degree confirmed that quercetin possessed the most potent targeted binding capacity toward SRC protein. It is speculated that quercetin may occupy the key functional sites of the SRC protein, thereby modulating its kinase activity and regulating the downstream signaling pathway transduction. This provides one of the core material bases for the antioxidant biological activity of litchi seed extract.

## 3. Discussion

This study took litchi seed extract as the research object. By establishing a D-galactose-induced oxidative stress mouse model and combining UPLC-MS, network pharmacology, and molecular docking, we systematically clarified the antioxidant activity of litchi seed extract and its underlying pharmacodynamic material basis. The findings provide solid scientific evidence and experimental support for the high-value utilization of litchi processing by-products and the development of natural antioxidant functional products.

The D-galactose-induced oxidative stress mouse model is a classic animal model for evaluating the antioxidant activity of natural products [21]. Its core mechanism is described as follows: D-galactose is converted into galactitol via aldose reductase catalysis, leading to cellular osmotic imbalance. Meanwhile, it consumes substantial nicotinamide adenine dinucleotide phosphate (NADPH), suppresses glutathione (GSH) regeneration, and disrupts the homeostasis of the endogenous antioxidant defense system. This further triggers mitochondrial dysfunction and excessive accumulation of ROS, ultimately causing oxidative damage to biological macromolecules, including proteins, lipids, and DNA. This pathological process closely resembles oxidative stress occurring during natural aging and under pathological conditions such as diabetes, fatty liver, and neurodegenerative diseases [22,23,24]. MDA is a terminal product of lipid peroxidation, and its content directly reflects the extent of oxidative injury [25]. SOD is a pivotal antioxidant enzyme that scavenges superoxide anion radicals (O_2_^−^), thereby maintaining the dynamic balance between ROS production and clearance [26,27]. This study revealed that compared with blank control mice, model mice displayed markedly elevated MDA content and compensatory upregulation of SOD activity in serum, liver, and kidney tissues, confirming the successful establishment of a D-galactose-induced oxidative stress model. After intervention with litchi seed extract, MDA levels in mouse serum, liver, and kidney decreased in a dose-dependent manner, while SOD activity was moderately increased. Moreover, vitamin E exhibited slightly stronger antioxidant capacity than litchi seed extract. Litchi seed extract alleviates oxidative stress through two major pathways: lowering the level of lipid peroxidation and enhancing antioxidant enzyme activities. It can relieve oxidative stress-related damage by reinforcing the endogenous antioxidant defense system and scavenging excess free radicals.

Plant extracts possess complex chemical compositions. Most constituents exert pharmacological effects only after absorption into the blood via the gastrointestinal tract; thus, blood-entering ingredients are regarded as the actual pharmacodynamic material basis. For analyzing the blood-entering constituents of litchi seed extract, a mouse oxidative stress model, rather than normal mice, should be employed for the following reasons: (1) Pathological conditions can alter the physiological microenvironment of the organism, which may affect the absorption, transport, metabolism, and excretion of plant extracts. This leads to differences in the types and concentrations of blood-entering constituents compared with the physiological state [28,29]. (2) Data on blood-entering constituents obtained from disease models can more accurately reflect the in vivo characteristics of the extract, facilitating the identification of key active components and clarification of its underlying mechanism of action in the organism. In comparison, results derived from normal mice may deviate substantially from real pathological conditions [30]. (3) Disease status may also influence the toxic response to the extract. Investigation of blood-entering constituents based on disease models therefore has higher clinical reference value than studies using normal mice [31,32]. Section 2.2 demonstrated that long-term high-dose D-galactose injection successfully induces oxidative stress in mice. Accordingly, this identical model was adopted in the current study to analyze the blood-entering constituents of litchi seed extract. Based on serum pharmacochemistry theory, UHPLC-MS was applied to systematically characterize the chemical profiles of litchi seed extract and drug-containing serum collected from mice after intragastric administration. By comparing the total ion current chromatograms of blank serum, raw extract, and drug-containing serum, a total of 13 prototype blood-entering constituents were identified, comprising 8 flavonoids, 3 organic acids, and 2 polyphenols. These constituents included gallic acid, citric acid, L-tyrosine, epicatechin, quercetin, protocatechuic acid, gallocatechin, epicatechin gallate, cinnamtannin B1, isorhamnetin, diosmetin, naringin, and naringenin. All of these components are small-molecule compounds absorbable into the bloodstream via the gastrointestinal tract, and may act as the direct pharmacodynamic material basis for the antioxidant effects of litchi seed extract. This strategy overcomes the limitations of traditional in vitro component analysis, which cannot reflect the actual effective constituents in vivo. Among the 13 prototype blood-entering constituents, flavonoids accounted for 61.5% of the total, further verifying that flavonoids represent the core functional components underlying the antioxidant activity of litchi seeds. This result is consistent with the findings of total flavonoid content quantification.

To elucidate the material basis responsible for the antioxidant activity of litchi seed extract, thirteen prototype components absorbed into the blood were selected as the research subjects. A total of 183 functional targets were closely associated with these candidate compounds and antioxidant bioactivity. Further network topological analysis revealed that isorhamnetin, quercetin, naringenin, and diosmetin are the major core active constituents contributing to the antioxidant effects. Previous studies have confirmed that the four isolated compounds from litchi seed exert remarkable antioxidant capacities in multiple experimental models [33,34,35,36]. For instance, flavonoid compounds such as naringenin were verified to enhance the activities of SOD, catalase, and glutathione peroxidase in *Caenorhabditis elegans*, thereby elevating the overall antioxidant level of organisms [35]; diosmetin could reduce intracellular ROS content by 13.53–15.18% and increase SOD activity by 104.67% under oxidative stress conditions [36]. A PPI network of potential target genes was constructed using the STRING database, from which six core genes closely associated with antioxidant activity were screened: *IGF1R*, *PIK3R1*, *SRC*, *PIK3CA*, *ERBB2*, and *EGFR*. Cumulative studies have confirmed that these genes serve as master regulators of oxidative stress responses and intracellular signal transduction [37,38,39,40,41,42]. Studies show that suppressed *IGF1R* expression significantly improves oxidative stress resistance in mice; heterozygous *IGF1R* knockout mice exhibit a 26% longer average lifespan [37]. Mechanistic research indicates that PIK3R1 protein mediates redox balance via S-glutathionylation and controls autophagic flux to modulate cellular oxidative stress levels [38]. Additional functional research reports that activated PIK3CA and EGFR proteins induce robust antioxidant cascades by upregulating antioxidant enzymes, including MnSOD [39]. Taken together, published evidence suggests that litchi seed exerts antioxidant bioactivity by modulating these core targets and their downstream signaling pathways. The SRC kinase protein serves as both a redox sensor and a downstream effector in cellular redox signaling cascades. ROS, H_2_O_2_ produced by NADPH oxidases (NOX), dynamically modulate the catalytic activity of SRC through reversible oxidative modification of conserved cysteine residues [43]. H_2_O_2_-dependent sulfenylation at the Cys-185 and Cys-277 sites disrupts the intramolecular autoinhibitory complex formed by pTyr-527 and the SH2 domain. This conformational rearrangement relieves SRC autoinhibition and facilitates autophosphorylation at the Tyr-416 activation loop [43,44]. Such ROS-mediated SRC activation creates a reciprocal positive feedback loop that coordinates with NOX-driven ROS generation [44]. Consequently, SRC acts as a core signaling intermediate capable of sensing and propagating redox signals to regulate cell migration. Molecular docking validation showed that the binding energies of the four core components to the SRC protein were all lower than −7.0 kcal/mol, indicating favorable spontaneous and stable binding affinity. Quercetin displayed the lowest binding energy of −8.8 kcal/mol and formed strong polar interactions with key amino acid residues of the SRC protein. These results confirm that the core active components can target and bind to the SRC protein at the molecular level, further verifying the reliability of the network pharmacology screening results.

This study also has several limitations. Only blood-entering prototype components were identified, while their metabolites—which may also serve as important active substances—were not analyzed. In addition, in vitro and in vivo functional validation of core targets was not performed, and the antioxidant mechanism remains to be further elucidated. Future research will focus on systematic analysis of the in vivo metabolites of litchi seed constituents to complete the profile of its pharmacodynamic material basis. Further studies will also adopt approaches such as gene knockout, gene overexpression, and target inhibitors to validate the functions of core targets, including *SRC*, so as to clarify the molecular mechanism underlying the antioxidant action of litchi seed extract.

## 4. Materials and Methods

### 4.1. Materials and Chemicals

The Feizixiao litchi fruits applied in the present study were collected from an agricultural ecological garden in Lingshan County, Guangxi Zhuang Autonomous Region. Simple random sampling was adopted for sample collection. The sample size was rationally determined in accordance with statistical methods and pre-experimental results to ensure the reliability of experimental data. Taxonomic identification was carried out by Prof. Weibin Xu (Herbarium Institute) based on morphological characteristics, referring to Flora of China. A voucher specimen (No. 2024-8-12) was deposited in the Herbarium of Guangxi Institute of Botany. Eight-week-old SPF-grade C57BL/6 mice were purchased from Hunan Slack Jingda Experimental Animal Co., Ltd. (Changsha, China), with the animal production license certificate No. SCXK (Xiang) 2021-0002. The mouse feed was also provided by Hunan Slack Jingda Experimental Animal Co., Ltd. (Changsha, China), and its production license number was SCXK (Xiang) 2024-0007.

### 4.2. Preparation of Litchi Seed Extract

This extraction procedure was modified from a previously reported protocol [2]. Mature litchi fruits were peeled to remove the pericarp, pulp, and aril. The isolated litchi seeds were rinsed thoroughly and air-dried, then further dried in an oven at 60 °C to constant weight. The dried seeds were ground with a pulverizer and passed through a 40-mesh sieve to obtain fine powder. Subsequently, 200.00 g litchi seed powder was soaked in 95% ethanol for 12 h, followed by ultrasonic extraction for 20 min. The supernatant was filtered and concentrated to yield a crude extract. The residual solid was re-soaked and extracted three times in parallel, and all filtrates were collected and concentrated separately. All obtained extracts were pooled together. The combined extract was dissolved in water and loaded onto an XDA-7 macroporous resin column. The column was first rinsed with deionized water to eliminate water-soluble pigments, and then eluted with 60% ethanol solution to collect the target eluate. The eluate was concentrated under reduced pressure using an N-3100 rotary evaporator (Eyela, Tokyo, Japan) at a vacuum of 95 kPa, heating bath temperature of 40 °C, and cooling water temperature of 15 °C. The concentrated solution was further dried with an HF-015 spray dryer (Hefan, Shanghai, China) to finally obtain 14.23 g of litchi seed extract.

### 4.3. Determination of Total Flavonoid Content in Litchi Seed Extract

The total flavonoid content of litchi seed extract was determined via the aluminum chloride colorimetric method according to a previously reported procedure [19]. Rutin standard solutions at concentrations ranging from 0 to 50 μg/mL were prepared with ethanol. Briefly, 0.2 mL of each rutin standard solution was mixed with 0.04 mL of 0.1 M potassium acetate solution, 0.6 mL of absolute ethanol, 1.8 mL of distilled water, and 0.04 mL of 10% (*w*/*v*) aluminum chloride solution. The mixture was incubated in the dark at 24 °C for 0.5 h. After reaction, the absorbance was measured at 415 nm using a T6 spectrophotometer (Tongyong, Beijing, China). A standard curve was established by linear regression, with rutin concentration as the abscissa (x-axis) and absorbance (OD value) as the ordinate (y-axis). Litchi seed extract solution (0.1 mg/mL) was prepared with 50% ethanol and determined following the same protocol. All samples were analyzed in triplicate, and the mean value was calculated. The total flavonoid content was expressed as milligrams of rutin equivalent per gram of litchi seed extract (mg RE/g).

### 4.4. Study on the In Vivo Antioxidant Effect of Litchi Seed Extract

#### 4.4.1. Design of Animal Experiment

All animal experimental procedures were approved by the Research Ethics Committee of Guangxi Institute of Botany, Guangxi Zhuang Autonomous Region and Chinese Academy of Sciences (approval No. GXZW-2025041001). After 7 days of adaptive feeding, C57 mice were randomly divided into five groups with 12 mice per group: blank control group (BCG), model control group (MCG), positive control group (PCG), low-dose litchi seed extract group (L-LSG), and high-dose litchi seed extract group (H-LSG). Mice in the PCG were treated with vitamin E at 50 mg/kg body weight. The L-LSG and H-LSG were administered litchi seed extract at doses of 100 mg/kg and 200 mg/kg body weight, respectively. The BCG and MCG received an equal volume of distilled water. All treatments were administered to mice via intragastric gavage once daily for 8 consecutive weeks. Meanwhile, except for the BCG, all mice were subcutaneously injected with D-galactose at 400 mg/kg body weight for 8 consecutive weeks [19]; the BCG was injected with an equal volume of normal saline. Body weight was recorded weekly, and the administration dosage was adjusted accordingly.

#### 4.4.2. Handling of Experimental Animals [45]

After 8 consecutive weeks of intervention, mice were anesthetized with sodium pentobarbital. Upon complete loss of consciousness, blood samples were collected from the retroorbital venous plexus, and the mice were then euthanized by cervical dislocation. Subsequently, liver and kidney tissues were rapidly excised and dissected. Collected blood samples were immediately transferred into sterile Eppendorf tubes and centrifuged at 1000× *g* for 10 min at 4 °C to isolate serum. The separated serum was stored at −20 °C until use. Equal portions of liver and kidney tissues from each mouse were homogenized with a glass homogenizer in cold phosphate-buffered saline (PBS, pH 7.4) at a tissue-to-buffer weight/volume ratio of 1:9 (g/mL). The tissue homogenates were centrifuged at 3500× *g* for 10 min at 4 °C, and the supernatants were collected and stored at −80 °C for subsequent biochemical assays.

#### 4.4.3. Determination of Antioxidant-Related Biochemical Indices

Protein concentrations in liver and kidney tissues were determined using a commercial assay kit (Beyotime, Shanghai, China). The activities of SOD and levels of MDA in liver, kidney, and serum were determined using matching assay kits (Jiancheng, Nanjing, China).

### 4.5. Analysis of Blood-Entering Prototype Constituents of Litchi Seed Extract

#### 4.5.1. Identification of Chemical Constituents in Litchi Seed Extract

Chemical constituents in litchi seed extract were analyzed by UHPLC-MS. The instrumental platform consisted of an ultra-high performance liquid chromatography system coupled to a high-resolution mass spectrometer (both Thermo Fisher Scientific, Waltham, MA, USA). Raw MS data were processed using Progenesis QI software (version 3.0) (Waters Corporation, Milford, MA, USA). Compound identification was carried out according to MS^2^ fragmentation patterns, with reference to the PubChem (https://pubchem.ncbi.nlm.nih.gov/) and ChemicalBook (https://www.chemicalbook.com/) databases.

#### 4.5.2. Treatment of Experimental Mice

Twelve mice were used to establish the oxidative stress model according to the procedures described in Section 4.4.1. After successful model establishment, the mice were randomly divided into a control group and an administration group. The administration group was intragastrically given litchi seed extract at 500 mg/kg, while the control group received an equal volume of distilled water, once daily for 3 consecutive days. Following the final administration, mice were anesthetized via isoflurane inhalation using an anesthesia machine. Blood samples were collected from the orbital venous plexus at 15, 30, 60, 120, and 180 min post-dosing. Samples were allowed to stand for 30 min and then centrifuged to harvest the supernatant. At each time point, 100 μL of supernatant was collected, and all aliquots were pooled into a single tube to obtain a total volume of 500 μL. The combined sample was immediately snap-frozen in liquid nitrogen and stored at −80 °C for subsequent analysis.

#### 4.5.3. Pretreatment of Serum Samples

Mouse serum samples stored at −80 °C were removed and thawed slowly on ice. An aliquot of 150 μL serum was transferred into a 1.5 mL Eppendorf tube, and 450 μL of methanol–acetonitrile (2:1, *v*/*v*) was added for protein precipitation. The mixture was vortexed for 1 min, ultrasonicated in an ice–water bath for 10 min, and then kept at −40 °C for 30 min. After centrifugation at 11,900× *g* for 10 min at 4 °C, 500 μL of the supernatant was transferred to an autosampler vial and evaporated to dryness. The dried residue was reconstituted with 150 μL of water–methanol–acetonitrile (1:2:1, *v*/*v*/*v*), vortexed for 1 min, and ultrasonicated for 3 min. The solution was stored at 4 °C overnight and centrifuged again at 11,900× *g* for 10 min at 4 °C. Finally, 100 μL of the supernatant was collected for subsequent UHPLC-MS analysis.

#### 4.5.4. Identification of Prototype Components in Mouse Serum

The components in mouse serum were analyzed using the same UHPLC-MS method described above for chemical constituent profiling of litchi seed extract. By comparing the TIC chromatograms of litchi seed extract, drug-containing serum, and blank serum, a compound was identified as a blood-absorbed prototype component if it was detected in both litchi seed extract and drug-containing serum, but absent in blank serum.

### 4.6. Identification of Antioxidant Active Components in Litchi Seed Extract via Network Pharmacology

#### 4.6.1. Target Acquisition for Blood-Entering Prototype Constituents of Litchi Seed Extract

Blood-entering prototype constituents of litchi seed extract were selected as the research objects. Their molecular structures were retrieved from the ChemSpider database (https://www.chemspider.com/) and saved in SMILES format. The structural files were then imported into SwissTargetPrediction (https://swisstargetprediction.ch/) for potential protein target prediction. Only targets with a predicted probability greater than 0 were retained. All candidate targets were consolidated, and duplicate entries were eliminated to construct a non-redundant target dataset for subsequent network pharmacology analysis.

#### 4.6.2. Retrieval of Antioxidant-Associated Genes

Antioxidant-related disease targets were retrieved from the GeneCards (https://www.genecards.org/), OMIM (https://www.omim.org/), and NCBI Gene (https://www.ncbi.nlm.nih.gov/gene/, accessed on 18 March 2026) databases using the keywords “antioxidant”, “antioxidative”, and “antioxidation”, with the species restricted to *Homo sapiens*. All retrieved targets were combined, and duplicate entries were removed to construct the final antioxidant-related target set.

#### 4.6.3. Component-Target Network Construction of Potential Antioxidant Components from Litchi Seed Extract

The intersection of litchi seed extract ingredient targets and antioxidant-related genes was identified as potential antioxidant targets using a Venn diagram drawn with Venny 2.1.0 (https://bioinfogp.cnb.csic.es/tools/venny/, accessed on 19 March 2026). The ingredient–overlapping target pairs were imported to construct “Network” and “Type” files. A component–target interaction network was built using Cytoscape 3.10.3 (Cytoscape Team, San Diego, CA, USA), and topological analysis was performed to extract key topological parameters of the network.

#### 4.6.4. PPI Network and Core Target Identification

PPI analysis of the overlapping targets was performed using the STRING database (https://cn.string-db.org/), with the species limited to *Homo sapiens* and the minimum interaction confidence score set at 0.9. Unconnected, isolated targets were hidden. The PPI network data were exported as a TSV file, and the network graph was saved in PNG format. The TSV file was then imported into Cytoscape 3.10.3 for topological analysis to obtain the key parametric characteristics of the overlapping target PPI network. The built-in MCODE plugin of Cytoscape 3.10.3 was applied to screen core hub targets, and the highest-scoring core target subnetwork was retrieved. Further topological analysis was carried out to acquire relevant data of the core subnetwork, and the PPI network of core targets was subsequently constructed.

#### 4.6.5. Molecular Docking

Molecular docking analysis was performed between high-ranking active components and key antioxidant-related target genes. The structures of active components were imported into Chem3D 19.0 (PerkinElmer Inc., Waltham, MA, USA). Energy minimization was carried out using the MM2 module to generate the lowest-energy optimal conformation, which was saved in MOL2 format and further converted to PDBQT format via MGLTools 1.5.7 (The Scripps Research Institute, La Jolla, CA, USA). Three-dimensional structures of human target proteins (Homo sapiens) were retrieved from the UniProt database (https://www.uniprot.org/) and saved as PDB files. Hydrogen atoms were added in MGLTools 1.5.7; proteins were defined as receptors and exported as PDBQT files, followed by identification of protein active pockets using the grid box function. Ligand–receptor molecular docking was implemented with the AutoDock Vina plugin (version 2.1.0), and docking results were visualized using PyMOL 3.2 (Schrödinger, New York, NY, USA).

### 4.7. Data Statistics

Two batches of animal experiments were carried out with C57BL/6 mice. The first experiment contained five groups with 12 mice in each group for antioxidant activity evaluation, while the second experiment included two groups with 6 mice per group for the analysis of blood-entering constituents. All data were expressed as mean ± standard deviation (SD). Statistical analyses were performed via GraphPad Prism 8. One-way ANOVA was selected as the statistical model for intergroup comparison, and Tukey’s post hoc test was further used for pairwise analyses. A *p* value of less than 0.05 was defined as statistically significant.

## 5. Conclusions

This study validated the strong in vivo antioxidant capacity of litchi seed extract using a D-galactose-induced mouse oxidative stress model. Biochemical detection results demonstrated that the extract could significantly decrease MDA content and increase SOD activity in mouse serum, liver, and kidney tissues, indicating its protective effect against oxidative damage. Thirteen prototype constituents absorbed into blood were successfully identified via UHPLC-MS analysis, among which flavonoids accounted for the largest proportion and were regarded as the primary active components of litchi seed extract. Further network pharmacology screening combined with molecular docking verification revealed that four flavonoid monomers, namely isorhamnetin, quercetin, naringenin, and diosmetin, could bind stably to the core hub protein SRC with favorable binding affinity. Collectively, by integrating serum pharmacochemistry, high-resolution mass spectrometry, network pharmacology, and molecular docking, this study systematically illustrated the active components, key targets, and potential molecular mechanism responsible for the antioxidant effect of litchi seed extract.

## Figures and Tables

**Table 1:** Total flavonoid content of litchi seed extract.

Item	Litchi Seed Extract
Total polyphenols (%)	68.37 ± 1.98

Values are mean of triplicate experiments ± standard deviation.

**Table 2:** Detection of blood-entering prototype components of litchi seed extract.

No.	Retention Time (min)	Mass-to-Charge Ratio	Ion	Formula	Identification	Peak Areas (Extract)	Peak Areas (Drug Administration)	Peak Areas (Blank Control)
1	0.770	173.1044	M−H	C_6_H_14_N_4_O_2_	D-Arginine	943,388.81	54,590.34	24,020.89
2	0.867	105.0192	M−H	C_3_H_6_O_4_	L-glyceric acid	116,197.61	16,119.28	15,315.08
3	1.198	387.0057	M−H	C_12_H_22_O_11_	Sucrose	18,765.01	0	0
4	1.872	171.1033	M+H	C_7_H_6_O_5_	Gallic acid	13,470.46	4437.85	0
5	2.143	191.0197	M−H	C_10_H_6_O_3_	Citric acid	157,939.67	110,433.05	0
6	2.254	180.0991	M+H	C_9_H_11_NO_3_	L-Tyrosine	28,636.35	23,944.68	0
7	2.463	291.0863	M+H	C_15_H_14_O_6_	Epicatechin	6706.57	3408.15	0
8	2.879	195.0771	M−H	C_10_H_12_O_4_	Paeonilactone B	33,086.05	2607.23	1379.08
9	4.526	375.1309	M−H	C_16_H_24_O_10_	Loganic acid	46,690.02	0	0
10	4.763	487.1468	M−H	C_21_H_28_O_13_	Cistanoside F	19,747.04	0	0
11	5.337	301.0769	M−H	C_15_H_10_O_7_	Quercetin	10,484.64	3635.78	0
12	5.481	209.0991	M−H	C_7_H_6_O_4_	Protocatechuic acid	70,295.35	13,065.07	0
13	5.789	449.1465	M−H	C_22_H_26_O_10_	Forsythenside A	23,673.40	0	0
14	5.873	609.1803	M+H	C_28_H_32_O_15_	Spinosin	174,919.35	0	0
15	5.997	305.0334	M−H	C_15_H_14_O_7_	Gallocatechin	14,078.54	11,688.44	0
16	6.120	441.0840	M+H	C_22_H_18_O_10_	Epicatechin Gallate	49,468.38	9652.85	0
17	6.393	579.1753	M−H	C_27_H_32_O_14_	Naringin	33,322.65	0	0
18	6.422	785.2273	M+H	C_38_H_40_O_18_	6-Feruloylspinosin	40,797.26	0	0
19	6.520	433.1120	M+H	C_21_H_20_O_10_	Spinosin6	25,637.46	0	0
20	7.174	865.1794	M+H	C_45_H_36_O_18_	Cinnamtannin B1	17,836.03	8469.61	0
21	7.397	473.1098	M−H	C_23_H_22_O_11_	6-O-Acetylisovitexin	19,436.72	0	0
22	7.544	287.0544	M+H	C_15_H_10_O_6_	Luteolin	26,534.04	0	0
23	7.711	317.0194	M+H	C_16_H_12_O_7_	Isorhamnetin	92,777.12	15,767.30	0
24	8.054	177.0542	M+H	C_10_H_8_O_3_	7-Methoxycoumarin	8346.92	0	0
25	8.310	301.0699	M+H	C_16_H_12_O_6_	Diosmetin	16,097.49	6047.70	0
26	9.484	315.0852	M+H	C_17_H_14_O_6_	Velutin	27,979.71	0	0
27	10.408	579.1097	M−H	C_27_H_32_O_14_	Naringin	71,913.05	5106.76	0
28	12.218	337.1450	M−H	C_21_H_22_O_4_	8-Geranyloxypsoralen	16,868.57	0	0
29	12.777	271.2889	M−H	C_15_H_12_O_5_	Naringenin	9954.49	6986.18	0
30	14.110	758.5680	M+H	C_42_H_80_NO_8_P	Lecithin	503,777.15	326,109.68	503,795.91
31	15.096	108.0808	M+H	C_7_H_9_N	3-ethylpyridine	11,109.12	0	0

**Table 3:** Molecular Docking Binding Energy Evaluation.

Protein	Ligand	Docking Score (kcal/mol)
SRC	Diosmetin	−7.3
SRC	Naringenin	−7.3
SRC	Isorhamnetin	−8.2
SRC	Quercetin	−8.8

## Data Availability

Data are contained within the article.

## Abbreviations

The following abbreviations are used in this manuscript:

BCG	Blank control group
DPPH	1,1-diphenyl-2-picrylhydrazyl
*ERBB2*	Erb-B2 receptor tyrosine kinase
*EGFR*	Epidermal growth factor receptor
GSH	Suppresses glutathione
H-LSG	High-dose litchi seed extract group
*IGF1R*	Insulin-like growth factor 1 receptor
L-LSG	Low-dose litchi seed extract group
MCG	Model control group
MDA	Malondialdehyde
NADPH	Nicotinamide adenine dinucleotide phosphate
NOX	NADPH oxidases
PCG	Positive control group
*PIK3CA*	Phosphoinosi-tide-3-kinase catalytic subunit alpha
*PIK3R1*	Phosphoinositide-3-kinase regulatory subunit 1
PPI	Protein–protein interaction
ROS	Reactive oxygen species
SOD	Superoxide dismutase
*SRC*	Proto-oncogene tyrosine-protein kinase Src
TCM	Traditional Chinese Medicine
TIC	Total ion current
UHPLC-MS	Ultra-high performance liquid chromatography–mass spectrometry
